# Research on energy-saving algorithm of HVAC multi-agent system consensus based on event-triggered mechanism

**DOI:** 10.1371/journal.pone.0337139

**Published:** 2025-11-21

**Authors:** Weilin Wu, Shaoxiong Shi, Meihuan Lin, Haixiao Gong, Jiakan Li

**Affiliations:** 1 Center for Applied Mathematics of Guangxi, College of Physics and Electronic Information, Guangxi Minzu University, Guangxi, China; 2 Guangxi Colleges and Universities Engineering Research Center for Multi-modal Information Intelligent Sensing, Processing and Application, Guangxi Minzu University, Guangxi, China; 3 Guangxi Key Laboratory of Machine Vision and Intelligent Control, Wuzhou University, Guangxi, China; 4 Guigang Information and Government Service Center, Guangxi, China; The Hong Kong Polytechnic University, HONG KONG

## Abstract

This paper designs an energy-saving algorithm for HVAC multi-agent systems based on event-triggered mechanism. In this algorithm, the incremental cost of the generator set and the incremental benefit of the flexible load are taken as the consistency variables, and the discrete time consistency algorithm is used to realize the distributed economic dispatch. The algorithm controls the on-demand transmission of information by designing a trigger control condition to avoid transmitting redundant information in the network, thereby reducing the network transmission pressure. In addition, this paper also discusses how to use graph theory to describe the communication topology between agents, and how to use consensus algorithm to analyze the stability and convergence of the system. This paper verifies that the designed trigger conditions are sent to the interacting neighbor generators on demand in the form of discrete unequal periods. The simulation results show that the incremental cost of all generators in the system eventually tends to the same value, and the optimization goal of economic dispatch is realized. At the same time, the trigger time of each generator is discrete and unequal cycle, which proves that the algorithm based on event trigger mechanism can determine whether the control task is executed according to the preset trigger condition, so as to realize the on-demand execution.

## 1. Introduction

With the rapid development of artificial intelligence and network communication technology, multi-agent systems have rapidly become a research hotspot as its branch field, and have achieved rich research results [1–4]. Although event-triggered consensus algorithms have achieved many theoretical results, their application to HVAC systems in intelligent buildings is still very limited. Since HVAC accounts for more than 55% of building energy consumption, improving its energy efficiency is of great importance. Therefore, our motivation is to design a distributed consensus-based energy-saving algorithm tailored for HVAC systems under event-triggered mechanisms to reduce communication costs, save energy, and ensure stability. The existing research results of distributed consensus control have been widely applied to engineering fields, including formation control, networked control, and unmanned aerial vehicles [5–7]. In the 1970s, Degroot [8] first proposed the consistency problem in the study of management science and statistics. Consensus, as a basic problem in cooperative control, means that all agents in the system converge to a common value under the designed appropriate control protocol [9]. In recent years, the consensus problem of multi-agent systems has achieved fruitful results. Its theoretical research can be applied to many important fields, such as multi-robot systems [10], smart grid systems [11], intelligent transportation network systems [12], wireless sensor network systems [13].

In practical applications, a single agent of the system usually has limited computing resources and network bandwidth [14]. In the existing consensus control strategy, the agent needs to continuously detect its own state, continuously exchange information with its neighbors, and update the controller at high frequency. In order to save system resources and overcome the continuous communication between agents, Li et al. [15] proposed sampling control, in which information transmission and controller update are only performed at the sampling time. However, when the system state changes are small, the update of the controller is not necessary, so the sampling control will still cause a waste of resources. To address this issue, Astrom et al. [16] proposed an event-triggered mechanism (ETM) for random systems and demonstrated that this mechanism offers superior energy efficiency compared to time-triggered mechanisms. Subsequently, Shoukry [17] employed this mechanism to control the networked system, achieving favourable results in terms of resource utilisation efficiency. Different from the time-triggered mechanism, under ETM, the execution of the control operation depends on the event-triggered condition of the system state, and the time interval of the trigger sequence of the determined control operation is time-varying and aperiodic. By considering the actual situation of the system and reducing unnecessary control operations, ETM can greatly reduce the resource consumption of the system. Therefore, it is of great theoretical and practical value to design a distributed cooperative consensus algorithm combined with ETM, taking into account the communication cost between agents and the update cost of the controller [18].

In this control mechanism, the specific event that the system measurement error exceeds the specified threshold has been widely used in engineering. That is, when the measured value reaches a preset threshold, one or more events are triggered, which trigger a series of actions such as inter-agent interaction [19]. A lot of research results have been achieved by applying the event-triggered mechanism to different control fields, such as state feedback and output feedback control [20], tracking control [21], and control with quantized systems [1]. In 2009, Dimarogaoas et al. [22] introduced centralized and distributed event-triggered controllers for the first time in the consensus problem of multi-agent systems. For second-order systems, Xu et al. [23] explored the consensus problem for time-varying reference signals. Dai et al. [24] proposed a scheme utilising slave information to design a distributed event-triggered controller, addressing the leader-tracking consensus problem when leader information is unknown. In [25,26], event-triggering is introduced into homogeneous high-order linear systems. The event-triggering study of heterogeneous high-order systems includes the study of nonlinear systems, such as Lagrangian systems. In general, the error design is the difference between its own state and the state at the trigger moment [27]. Su et al. [25] designed a controller based on combined sampling error, incorporating both each agent’s information at the trigger moment and its neighbours’ instantaneous information at that moment. They proposed a self-triggering mechanism to address the consistency issue. The self-triggered mechanism is actively completed relative to the event-triggered [28], that is, the next update time of the self-triggered control is to pre-calculate the controller update time based on the dynamic previously received data and information. Using this combined type error design, the event-triggered controller designed in [29], and the output regulation problem.

It is worth noting that HVAC (Heating, Ventilation, and Air Conditioning) accounts for more than 55% of building energy consumption in Southeast Asia [30]. Therefore, how to optimize the control strategy of the HVAC system at the building end, reduce energy consumption, and accelerating the realization of carbon neutrality is of great significance. At the same time, although the event-triggered mechanism has many scientific research results, the research results of event-triggered control for HVAC systems in intelligent buildings are very limited. Motivated by the practical need to reduce communication and computation overhead while guaranteeing stability and acceptable control performance in networked HVAC applications, this paper develops a distributed event-triggered consensus framework tailored to HVAC control at the building level. The proposed approach combines a carefully designed local event condition that accounts for HVAC-specific dynamics with a distributed consensus protocol to coordinate multiple controllers. The main contributions are as follows:

1) We propose a distributed event-triggered consensus algorithm with a hybrid error design that reduces unnecessary communication and controller updates while preserving closed-loop stability.
2) We provide rigorous stability and convergence analysis under the proposed triggering rules, and derive sufficient conditions that avoid Zeno behavior.
3) We validate the approach on representative HVAC models through numerical simulations (and, where available, hardware-in-the-loop/experimental tests), demonstrating reduced communication load and improved energy/control performance compared with time-triggered or naïve sampling baselines. These contributions address a gap in the existing literature by bridging event-triggered consensus theory and the practical requirements of building HVAC control.

Organization of the paper. The remainder of this paper is organized as follows. Section 2 presents preliminaries and problem formulation, including algebraic graph theory and the discrete-time multi-agent model. Section 3 introduces the proposed HVAC energy-saving algorithm based on an event-triggered mechanism and provides stability analysis. Section 4 gives numerical simulation examples that validate the effectiveness of the proposed method. Finally, Section 5 concludes the paper and discusses limitations and future work.

## 2. Preliminaries and problem formulation

### 2.1. Algebraic graph theory

The communication topology graph between agents is represented by a directed weighted graph, denoted as G=(V,ε,W) . Where V=(1,2,⋯,N) denotes the set of all vertices of the graph G, the directed edges of the graph G are denoted as $\varepsilon_{i,j}\subset V\times V=\left\{(i,j),i,j\in V\right\}$, and the set of neighbours of the pointi is denoted as $N_{i}$. $W=(w_{i,j})\in R^{N,N}$ denotes the adjacency matrix, where$w_{i,j}$ denotes the weight value of the edge $\varepsilon_{i,j}$. *When*$\varepsilon_{j,i}\in \varepsilon$, there is, otherwise, there is$w_{i,j}=0$. $w_{i,j}>0$ denotes that the agenti receive inf $w_{i,j}>0$ ormation from agent j and vice versa. $D=diag(d_{1},d_{2},\cdots ,d_{N})\in R^{N,N}$ denote the incidence matrix, where $d_{i}=\sum {\mathrm{\backslash nolimits}}_{i=1,i\neq j}^{N}w_{i,j}$. Let $B=diag\left\{b_{1},b_{2},...,b_{N}\right\}$ denote the information interaction matrix between leader and follower nodes. The Laplacian matrix of graph G is $L=D-W\in R^{N,N}$, where the elements satisfy $l_{i,j}=\sum_{j=1,i\neq j}^{N}w_{i,j},l_{i,j}=-w_{i,j},i\neq j$. If there is a sequence of edges in the directed graph starting at node i and ending at node j, then there is a directed path from i to j. A directed spanning tree exists if there is a root node in the graph and directed paths exist from that node to all other nodes. Furthermore, if a directed spanning tree exists for the graph G, then its Laplacian matrix has one eigenvalue of zero and all other eigenvalues contain positive real parts [30].

### 2.2. Description of the problem

Consider a multi-agent system consisting of N agents, whose communication topology is represented by a directed weighted graph G, where each agent can be regarded as a node in the graph G, and each agent satisfies the following dynamic model [0]:


$$\left\{ \;\;\;\begin{array}{c} {\hat{x}}_{i}(t)=v_{i}(t)\\ {\hat{v}}_{i}(t)=u_{i}(t) \end{array}\;\;\;\;\right.,\;i=1,2,...$$ (1)

Where $x_{i}(t)\in R$ is the displacement of the agent i, $v_{i}(t)\in R$ is the velocity of the agent i and $u_{i}(t)\in R$ is the control input.

By introducingh as the optimized sampling interval of the agent, the continuous system can be discretized as follows:


$$\left\{ \;\;\;\begin{array}{c} x_{i}((k+1)h)=x_{i}(kh)+hv_{i}(kh)+\frac{\text{1}}{\text{2}}h^{2}u_{i}(kh)\\ v_{i}((k+1)h)=v_{i}(kh)+hu_{i}(kh) \end{array}\;\;\;\;\;\right.,\;i=1,2,...$$ (2)

Where $x_{i}(kh)\in R$, $v_{i}(kh)\in R$ and $u_{i}(kh)\in R$ are the displacement, velocity and control inputs of the agenti att=kh respectively.

***Assumption 1*** For system (2), its Laplace matrix is assumed to be


$$L=\left(\;\;\;\begin{array}{cc} {}*20cL_{11} & L_{12}\\ L_{21} & L_{22} \end{array}\;\;\;\right)$$ (3)

where $L_{11}$,$L_{12}$,$L_{21}$ and $L_{22}\in R^{n\times n}$ are row sum zero matrices and the subnetwork corresponding to $L_{11}$,$L_{22}$ contains a directed spanning tree.

***Lemma 1*** If the directed graph corresponding to system (2) satisfies Assumption 1, then the algebraic and geometric reweights of its Laplace matrix are both 2.

***Definition 1*** If for any initial condition, satisfy:


$$\left\{ \;\;\;\begin{array}{c} \underset{k\to \infty }{\lim}\left\|x_{i}(kh)-x_{j}(kh)\right\|=0\\ \underset{k\to \infty }{\lim}\left\|v_{i}(kh)-v_{j}(kh)\right\|=0 \end{array}\;\;\;\;\right.,\;\;\forall i,j\in l_{s},s=1,2$$ (4)

Then the second-order discrete multi-intelligent body system asymptotically reaches coherence.

***Definition 2*** If for any initial condition, satisfy:


$$\left\{ \;\;\;\begin{array}{c} \underset{k\to \infty }{\lim}\left\|x_{i}(kh)-x_{l}^{0}(kh)\right\|=0\\ \underset{k\to \infty }{\lim}\left\|v_{i}(kh)-v_{l}^{0}(kh)\right\|=0 \end{array}\;\;\;\;\;\right.,\;\;l=1,2$$ (5)

where$x_{l}^{0}$,$v_{l}^{0}$ are the displacement and velocity of the leader, respectively. Then the second-order discrete multi-agent system asymptotically achieves consistent following of the leader by the follower.

### 2.3. Consensus of discrete second-order multi-agents

It is assumed that information interaction can be carried out between power components. Let $x=(x_{1},x_{2},...,x_{n}{)}^{T}$, $v=(v_{1},v_{2},...,v_{n}{)}^{T}$,$u(t)=(u_{1},u_{2},...,u_{n}{)}^{T}$,k≥1, to achieve consistency of the second order system, consider the following consistency algorithm.


u(kh)=βLx((k−1)h)−αLx(kh),k≥1 (6)

Where α,β are the parameters to be designed and u(0)=0, then there are x(h)=x(0),v(h)=v(0).

By agreement (6), the system can be rewritten in the following form:


$$\left\{ \;\;\;\begin{array}{c} x((k+1)h)=x(kh)+hv(kh)-\frac{\text{1}}{\text{2}}h^{2}[\alpha Lx(kh)-\beta Lx((k-1)h)nonumber\\ v((k+1)h)=v(kh)+h[-\alpha Lx(kh)-\beta Lx((k-1)h)] \end{array}\right.$$

Converted to matrix form there are

$(\;\;\;\begin{array}{c} x((k+1)hnonumber\\ v((k+1)h) \end{array}\;\;\;)=(\;\;\;\begin{array}{cc} {}*20cI_{n}-\frac{\alpha h^{2}L}{\text{2}} & I_{n}h\\ -\alpha hL & I_{n} \end{array}\;\;\;)(\;\;\;\begin{array}{c} x((k+1)h)\\ v((k+1)h) \end{array}\;\;\;)+(\;\;\;\begin{array}{cc} {}*20c\frac{\beta h^{2}L}{\text{2}} & 0_{n\times n}\\ \beta hL & 0_{n\times n} \end{array}\;\;\;)(\;\;\;\begin{array}{c} x((k-1)h)\\ v((k-1)h) \end{array}\;\;\;)$,

Setting $K((k+1)h)=(\begin{array}{cccc} {}*20cx((k+1)h) & v((k+1)h) & x(kh) & v(kh) \end{array})$, we have


K((k+1)h)=AK(kh) (7)

Where


$$K=(\;\;\;\begin{array}{cccc} {}*20cI_{n}-\frac{\alpha h^{2}L}{\text{2}} & I_{n}h & \frac{\beta h^{2}L}{\text{2}} & 0_{n\times n}\\ -\alpha hL & I_{n} & \beta hL & 0_{n\times n}\\ I_{n} & 0_{n\times n} & 0_{n\times n} & 0_{n\times n}\\ 0_{n\times n} & I_{n} & 0_{n\times n} & 0_{n\times n} \end{array}\;)$$

The characteristic polynomial of the matrix K can be expressed as $\det(\lambda I_{4n}-K)$.


$$\begin{array}{c} \det(\lambda I_{4n}-K)=\det(\;\;\;\begin{array}{cccc} {}*20c\lambda I_{n}-I_{n}+\frac{\alpha h^{2}L}{\text{2}} & -I_{n}h & -\frac{\beta h^{2}L}{\text{2}} & 0_{n\times n}\\ \alpha hL & \lambda I_{n}-I_{n} & \lambda I_{n} & 0_{n\times n}\\ -I_{n} & 0_{n\times n} & 0_{n\times n} & 0_{n\times n}\\ 0_{n\times n} & -I_{n} & 0_{n\times n} & \lambda I_{n} \end{array}\;\;\;)\\ \;\;\;\;\;\;\;\;\;\;\;\;\;=\det(\;\;\;\begin{array}{cc} {}*20c\lambda^{2}I_{n}-\lambda I_{n}+\frac{\alpha h^{2}L}{\text{2}}-\frac{\beta h^{2}L}{\text{2}} & -\lambda hI_{n}\\ \lambda \alpha hL-\beta hL & \lambda^{2}I_{n}-\lambda I_{n} \end{array}\;\;\;)\\ \;\;\;\;\;\;\;\;\;\;\;\;\;\;\;\;\;\;\;\;\;\;\;\;\;\;\;\;\;\;\;\;\;\;\;\;\;\;\;\;\;\;=\det(\lambda (\lambda^{3}I_{n}-(2-\frac{\alpha h^{2}L}{\text{2}})\lambda^{2}I_{n}+(1+\frac{\alpha -\beta }{\text{2}}h^{2}L)\lambda I_{n}-\frac{\beta hL}{\text{2}}))\\ \;\;\;\;\;\;\;\;\;\;\;\;\;\;\;\;\;\;\;\;\;\;\;\;\;\;\;\;\;\;\;\;\;\;\;=\prod_{i=1}^{n}(\lambda (\lambda^{3}-(2-\frac{\alpha h^{2}\mu_{i}}{\text{2}})\lambda^{2}+(1+\frac{\alpha -\beta }{\text{2}}h^{2}\mu_{i})\lambda -\frac{\beta h\mu_{i}}{\text{2}})) \end{array}$$

can be obtained from $\mu_{i}=0$ when there are $\lambda_{1}=\lambda_{2}=0$, $\lambda_{3}=\lambda_{4}=0$.

### 2.4. Correspondence analysis

***Theorem 1*** If Assumption 1 holds, a sufficient condition for system (1) to achieve consistency is that the following condition holds:

(i) $h<\frac{\text{2}}{\sqrt{\left(\alpha +\beta \right)}\mu_{i}}$.
(ii) $(1-\frac{(\alpha +\beta )Re\mathrm{\backslash nolimits}(\mu_{i})h^{2}}{\text{4}})(\frac{\left(3\beta -\alpha )Re\mathrm{\backslash nolimits}(\mu_{i}\right)}{\text{4}}-\frac{(\alpha +\beta beta{\left|\mu_{i}\right|}^{2}h^{2}}{\text{8}})-\frac{\beta^{2}{\left(Im\mathrm{\backslash nolimits}(\mu_{i})\right)}^{2}h^{2}}{\text{4}}>0$
(iii) $\begin{array}{c} \frac{(\alpha +\beta )Re\mathrm{\backslash nolimits}(\mu_{i})h^{2}}{\text{4}}{(\frac{\left(3\beta -\alpha )Re\mathrm{\backslash nolimits}(\mu_{i}\right)}{\text{4}}-\frac{(\alpha +\beta betaRe\mathrm{\backslash nolimits}(\mu_{i})h^{2}}{\text{8}})}^{2}.\\ -(1-\frac{(\alpha +\beta )Re\mathrm{\backslash nolimits}(\mu_{i})h^{2}}{\text{4}})\frac{{\left(\alpha -\beta \right)}^{2}{\left(Im\mathrm{\backslash nolimits}(\mu_{i})\right)}^{2}}{16}-(\frac{(3\beta +\alpha )\beta }{\text{8}}-\frac{{\left(\alpha +\beta \right)}^{2}\beta Re\mathrm{\backslash nolimits}(\mu_{i})h^{2}}{32})\\ \times \frac{(\alpha -\beta )\beta {\left|\mu_{i}\right|}^{2}{\left(Im\mathrm{\backslash nolimits}(\mu_{i})\right)}^{2}h^{4}}{\text{8}}-\frac{{\left(\alpha -\beta \right)}^{2}(\alpha +\beta )\beta {\left(Re\mathrm{\backslash nolimits}(\mu_{i})\right)}^{2}{\left(Im\mathrm{\backslash nolimits}(\mu_{i})\right)}^{2}h^{4}}{128}\\ +(1-\frac{(\alpha +\beta )Re\mathrm{\backslash nolimits}(\mu_{i})h^{2}}{\text{4}})\frac{(\alpha -\beta )\beta {\left(Im\mathrm{\backslash nolimits}(\mu_{i})\right)}^{2}}{\text{8}}(\frac{(5\beta -3\alpha )Re\mathrm{\backslash nolimits}(\mu_{i})h^{2}}{\text{4}}-\frac{(\alpha +\beta )\beta {\left(Re\mathrm{\backslash nolimits}(\mu_{i})\right)}^{2}h^{4}}{\text{8}})>0. \end{array}$

where $\mu_{i}(i=3,4,...,n)$ is the non-zero eigenvalue of the Laplace matrixL.

**Proof:**

Let


$$\begin{array}{cc} y(\lambda ) & =\prod_{i=1}^{n}(\lambda (\lambda^{3}-(2-\frac{\alpha h^{2}\mu_{i}}{\text{2}})\lambda^{2}+(1+\frac{\alpha -\beta }{\text{2}}h^{2}\mu_{i})\lambda -\frac{\beta h\mu_{i}}{\text{2}}))\\ & =\lambda^{n+2}(\lambda -1{)}^{4}\prod_{i=3}^{n}(\lambda^{3}-(2-\frac{\alpha h^{2}\mu_{i}}{\text{2}})\lambda^{2}+(1+\frac{\alpha -\beta }{\text{2}}h^{2}\mu_{i})\lambda -\frac{\beta h\mu_{i}}{\text{2}}), \end{array}$$

Let $g(\lambda )=\lambda^{3}-(2-\frac{\alpha h^{2}\mu_{i}}{\text{2}})\lambda^{2}+(1+\frac{\alpha -\beta }{\text{2}}h^{2}\mu_{i})\lambda -\frac{\beta h\mu_{i}}{\text{2}}$,i=3,...,n, then y(λ) can be written as:


$$y(\lambda )=\lambda^{n+2}(\lambda -1{)}^{4}\prod_{i=3}^{n}g(\lambda )$$ (8)

Introducing the bilinear transformation $\lambda =\frac{1+s}{1-s}$, one obtains

$\hat{g}(s)=4s^{3}+(4-(\alpha +\beta )\mu_{i}h^{2})s^{2}+2\beta \mu_{i}h^{2}s+(\alpha -\beta )\mu_{i}h^{2},i=3,4,...,n$.

$p(s)=\frac{\hat{g}(s)}{\text{4}}=s^{3}+(a_{i1}+jb_{i1})s^{2}+(a_{i2}+jb_{i2})s+a_{i3}+jb_{i3}$.

included among these


$$\begin{array}{c} a_{i1}=1-\frac{\alpha +\beta }{\text{4}}h^{2}Re\mathrm{\backslash nolimits}(\mu_{i}),b_{i1}=-\frac{\alpha +\beta }{\text{4}}h^{2}Im\mathrm{\backslash nolimits}(\mu_{i}),\\ a_{i2}=\frac{\beta }{\text{2}}h^{2}Re\mathrm{\backslash nolimits}(\mu_{i}),b_{i2}=\frac{\beta }{\text{2}}h^{2}Im\mathrm{\backslash nolimits}(\mu_{i}),\\ a_{i3}=\frac{\alpha +\beta }{\text{4}}Re\mathrm{\backslash nolimits}(\mu_{i}),b_{i3}=\frac{\alpha -\beta }{\text{4}}h^{2}Im\mathrm{\backslash nolimits}(\mu_{i}). \end{array}$$

Thus, the polynomial g(λ) is Schur stable if and only if p(s) is Hurwitz stable. p(s) is Hurwitz stable if and only if conditions (i), (ii), and (iii) hold.

## 3. HVAC energy saving algorithm based on event-triggered mechanism

From the perspective of distributed optimization, the consistency algorithm is applied, and the IC of the generator set and the IB of the flexible load are used as consistency variables. The economic dispatch problem is solved by distributed optimization. The local controller (embedded in each generator set and flexible load) updates its own IC or IB based on the neighbor’s IC or IB. Choose a ‘ main unit ‘ and ‘ main load ‘ to decide whether to increase or decrease the global IC and IB. When the total generation power of the generator is greater than the total demand power of the load, the global IC will decrease, and vice versa. When the total demand power of the load is greater than the total power of the generator, the global IB will increase, and vice versa.

It is assumed that the power generation cost function of the generator set and the power benefit function of the flexible load are both quadratic functions. The power generation cost function of the generator set is as follows:

For all the multi-agents in the system (2) i∈V, design the event triggering function y(t), a series of event triggering moments $t_{k}(k=0,1,...)$ can be obtained from y(t)=0, so that $x_{0}(t_{k}),x_{i}(t_{k})$ denotes the sampling state of the leader and the follower i at the time of $t_{k}$, respectively, and the following control protocol is given:


$$C_{i}(P_{Gi})=\alpha_{i}+\beta_{i}P_{Gi}+\gamma_{i}{P^{2}}_{Gi},i\in S_{G}$$ (9)

The power usage benefit function for flexible loads is as follows.


$$B_{j}(P_{Dj})=a_{j}+b_{j}P_{Dj}+c_{j}{P^{2}}_{Dj},i\in S_{D}$$ (10)

The economic dispatch problem is an optimization problem in which generators and flexible loads maximize the economic efficiency of the overall power system operation under the conditions of satisfying, a series of operating constraints, viz.


$$\begin{array}{c} \max\sum_{i=1}^{n}C_{i}(P_{Gi})-B_{j}(P_{Dj}),\\ S.T.\sum_{j=1}^{n}P_{Dj}-\sum_{i=1}^{n}P_{Gi}=0.\\ P_{Gi,\min}\leq P_{Gi}\leq P_{Gi,\max},i\in S_{G}\\ P_{Dj,\min}\leq P_{Dj}\leq P_{Dj,\max},i\in S_{D} \end{array}$$ (11)

Where $\alpha_{i},\beta_{i},\gamma_{i}$ is the constant term, primary coefficient and quadratic coefficient of the power generation cost function; $\alpha_{j},\beta_{j},\gamma_{j}$ is the constant term, primary coefficient and quadratic coefficient of the power consumption benefit function, respectively: $P_{Dj}$ is the demanded power of the flexible load j; $P_{Gi}$ is the output power of the genset i; $S_{G}$ is the set of generators; $S_{D}$ is the set of flexible loads; $P_{Gi,\min}$ and $P_{Gi,\max}$ are the minimum and maximum output power of the generators, respectively; $P_{Dj,\min}$ and $P_{Dj,\max}$ are the minimum and maximum input power of the flexible loads, respectively. Using the classical Lagrange multiplier method to solve, so that the input represents the Lagrange multiplier corresponding to the equation constraints, without considering the constraints (11), the above equation-constrained optimization problem can be transformed into.


$$\min\Gamma =-\sum_{j\in S_{D}}B_{j}(P_{Dj})+\sum_{i\in S_{G}}C_{i}(P_{Gi})+\lambda \left(\sum_{j\in S_{D}}P_{Dj}-\sum_{i\in S_{G}}P_{Gi}\right)$$ (12)

The optimality condition can be obtained by taking the partial derivation of the variables$P_{Gi}$,$P_{Dj}$ andλ as follows.


$$\frac{\partial C_{1}}{\partial P_{G1}}=\frac{\partial C_{2}}{\partial P_{G2}}=\cdots =\frac{\partial C_{m}}{\partial P_{Gm}}=\frac{\partial B_{1}}{\partial P_{D1}}=\frac{\partial B_{2}}{\partial P_{D2}}=\cdots =\frac{\partial B_{g}}{\partial P_{Dg}}=\lambda$$ (13)

That is, the optimal solution for economic dispatch is to make the generator’s IC equal to the flexible load’s IB, where m denotes the number of generators and g denotes the number of flexible loads.

It is assumed that all flexible loads and gensets are operating within their power constraints. In this consistency algorithm, IC of the genset and IB of the flexible loads are defined as follows.


$$\begin{array}{c} IC_{i}=\frac{\partial C_{i}(P_{Gi})}{\partial P_{Gi}}=\lambda_{i},i\in S_{G}.\\ IB_{i}=\frac{\partial B_{j}(P_{Dj})}{\partial P_{Dj}}=\lambda_{j},j\in S_{D}. \end{array}$$ (14)

Selecting IC and IB as the consistency variables, the consistency algorithm is applied to update the IC from the genset with the formula.


$$\lambda_{i}((k+1)h)=\lambda_{i}(kh)+h\lambda_{j}(kh)-\frac{\text{1}}{\text{2}}h^{2}[\alpha L\lambda_{i}(kh)-\beta L\lambda_{i}((k-1)h)]$$ (15)

The updated equation for the IB from the load is.


$$\lambda_{j}((k+1)h)=\lambda_{j}(kh)+h[-\alpha L\lambda_{i}(kh)-\beta L\lambda_{i}((k-1)h)]$$ (16)

In order to satisfy the power balance constraints in the power system Eq. (9) denotes the difference between the actual power demanded by the flexible loads and the actual output power of the generating unit byΔP:


$$\Delta P=\sum_{j\in S_{D}}P_{Dj}-\sum_{i\in S_{G}}P_{Gi}$$ (17)

The update equation for the IC of the main generating unit is given by


$$\lambda_{i}((k+1)h)=\lambda_{i}(kh)+h\lambda_{j}(kh)-\frac{\text{1}}{\text{2}}h^{\text{2}}[\alpha L\lambda_{i}(kh)-\beta L\lambda_{i}((k-1)h)]+\varepsilon \Delta P(i\in S_{G}$$

### The update formula for the main load is


$$\lambda_{j}((k+1)h)=\lambda_{j}(kh)+h[-\alpha L\lambda_{i}(kh)-\beta L\lambda_{i}((k-1)h)]+\varepsilon \Delta P,j\in S_{D}$$

Whereε is the convergence coefficient, a positive scalar, which is related to the convergence speed of the distributed optimization of the main generating units and main loads. The main generating unit or main load can be determined by “centrality” search, including degree centrality, eigenvector centrality, median centrality and compact centrality.

From equations (14):


$$\begin{array}{c} P_{Gi}=\frac{\lambda_{i}-\beta_{i}}{2\gamma_{i}},i\in S_{G},\\ P_{Dj}=\frac{\lambda_{j}-b_{j}}{2c_{j}},j\in S_{D}. \end{array}$$

In this way, the power constraint of the generating unit can be modified as follows.


$$P_{Gi}(k)=\left\{ \;\;\;\begin{array}{c} \frac{\lambda_{i}-\beta_{i}}{2\gamma_{i}},P_{Gi,\min}\leq \frac{\lambda_{i}-\beta_{i}}{2\gamma_{i}}\leq P_{Gi,\max}\\ P_{Gi,\min},\frac{\lambda_{i}-\beta_{i}}{2\gamma_{i}}\leq \lambda_{i,\min}\\ P_{Gi,\max},\frac{\lambda_{i}-\beta_{i}}{2\gamma_{i}}\geq \lambda_{i,\max} \end{array}\right.\;\;\;\;,\;\;i\in S_{G}$$

The power constraint for the flexible load can be modified as follows.


$$P_{Dj}(k)=\left\{ \;\;\;\begin{array}{c} \frac{\lambda_{j}-b_{j}}{2c_{j}},P_{Di,\min}\leq \frac{\lambda_{j}-b_{j}}{2c_{j}}\leq P_{Di,\max}\\ P_{Dj,\min},\frac{\lambda_{j}-b_{j}}{2c_{j}}\leq P_{Dj,\min}\\ P_{Dj,\max},\frac{\lambda_{j}-b_{j}}{2c_{j}}\geq \lambda_{j,\max} \end{array}\right.\;\;\;,\;\;i\in S_{D}$$

**Fig 1:**
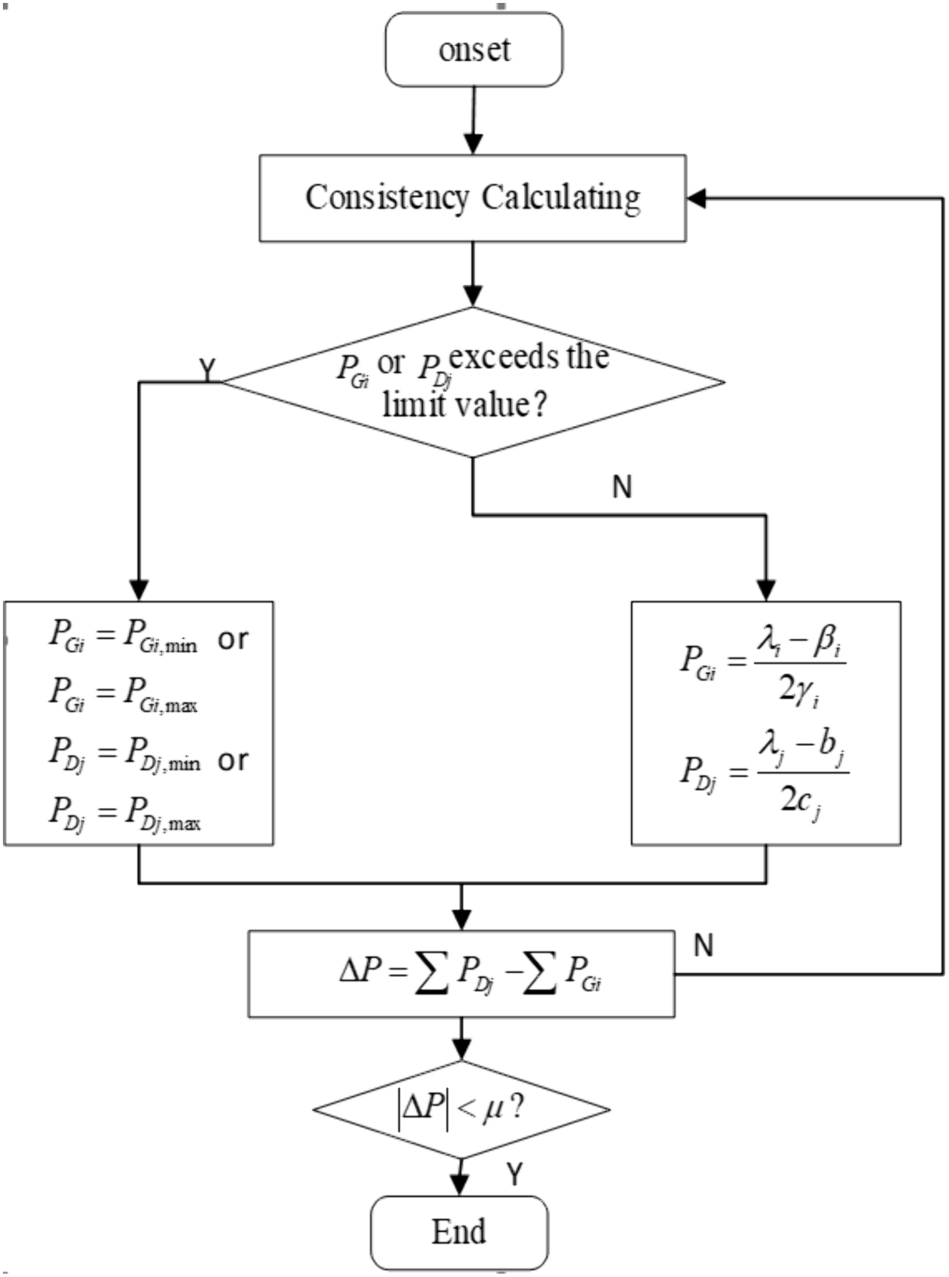
Algorithm Flow Chart.

The algorithm flow chart involved in the consistency-based distributed HVAC energy-saving algorithm is shown in Fig 1.

Based on graph theory and discrete-time consistency, a distributed scheduling based on an event-triggered mechanism is proposed. Firstly, it is stipulated that at the initial moment$t_{0}$, each generating unit in the power network exchanges information with its neighbors in the communication network and sends its respective incremental costs. At the same time, let$t_{i}^{0}=t_{0},i=1,2,3,...,n$, for the firsti power generation unit, mark its most recent trigger moment as$t_{i}^{k}$, wherek is the total number of trigger times of the power generation unit, if at the momentt satisfy the following trigger conditions:


$$\left\|{\hat{\lambda }}_{i}(k)-\lambda_{i}(k)\right\|>\frac{\beta {\left(\sum_{j=1}^{n}n_{ij}[\lambda_{i}(t_{i}^{k})-\lambda_{j}(t_{j}^{p(t)})]\right)}^{2}}{\left\|\left|N_{i}\right|\sum_{j=1}^{n}n_{ij}[\lambda_{i}(t_{i}^{k})-\lambda_{j}(t_{j}^{p(t)})]-\sum_{j=1}^{n}a_{ij}(\sum_{j=1}^{n}n_{jm}[\lambda_{j}(t_{j}^{p(t)})-\lambda_{m}(t_{m}^{p(t)})])\right\|}$$

Then mark this moment t as $t_{i}^{k+1}$.

Where 0<β<1, p(k) denotes the total number of triggers of the generating unit in front of the moment, $\left|N_{i}\right|$ denotes the number of neighbors that can communicate with the of the i generating unit, and $t_{m}^{p(t)}$ and $t_{j}^{p(t)}$ denote the most recent triggering moments of the m and j generating units in front of the moment t. The i generating unit stores its own trigger information $\lambda_{i}(t_{i}^{k+1})$ and passes it on to other generating units in the system that can communicate with it.

***Theorem 2***: For a discrete-time system, denoted by the incremental cost of generating units, the statex, all states in the system will reach unity if the triggering condition (5.9) is satisfied and the communication topology graph is strongly connected and undirected.

*Proof:* For the discrete-time consistent algorithm, expressing the above state in terms of the incremental cost of generating unitsx, one obtains.


$${\dot{\lambda }}_{i}(k)=-\sum_{j=1}^{n}a_{ij}(\lambda_{i}(t_{i}^{k})-\lambda_{j}(t_{i}^{k}))$$

Let $e_{i}(k)=\lambda_{i}(k)-\lambda_{i}(t_{i}^{k})$ denote the difference between the updated state value at the latest moment and the state value at this moment, in order to ensure the asymptotic stability of the system, a Liaplov function is introduced, which is defined as follows.


$$V(k)=\frac{\text{1}}{\text{2}}\lambda^{T}(k)L\lambda (k)$$

The first order partial derivative of this is obtained as


$$\dot{V}(k)=-e_{i}^{T}(k)LL\lambda (t_{i}^{k})-\lambda^{T}(t_{i}^{k})LL\lambda (t_{i}^{k})$$

honorific title


$$s_{i}(k)=\sum_{j=1}^{n}a_{ij}(\lambda_{j}(t_{i}^{k})-\lambda_{i}(t_{i}^{k}))$$

imitate


$$\dot{V}(k)=\sum_{i=1}^{n}e^{T}(k)\sum_{j=1}^{n}\left(s_{j}(k)-s_{i}(k)\right)-\sum_{i=1}^{n}{s_{i}}^{2}(k)$$

From the triggering condition, we have


$$\left\|\lambda_{i}(k)-\lambda_{i}(t_{i}^{k})\right\|\leq \frac{\beta {\left(\sum_{j=1}^{n}n_{ij}[\lambda_{i}(t_{i}^{k})-\lambda_{j}(t_{j}^{p(t)})]\right)}^{2}}{\left\|\left|N_{i}\right|\sum_{j=1}^{n}n_{ij}[\lambda_{i}(t_{i}^{k})-\lambda_{j}(t_{j}^{p(t)})]-\sum_{j=1}^{n}a_{ij}(\sum_{j=1}^{n}n_{jm}[\lambda_{j}(t_{j}^{p(t)})-\lambda_{m}(t_{m}^{p(t)})])\right\|}$$

$$\begin{array}{c} \dot{V}(t)\leq \sum_{i=1}^{n}\left|e^{T}(k)\right|\left|\sum_{j=1}^{n}\left(s_{j}(k)-s_{i}(k)\right)\right|-\sum_{i=1}^{n}{s_{i}}^{2}(k)\\ \leq (\beta -1){s_{i}}^{2}(t) \end{array}$$

From the above analysis it can be concluded that V(k)≥0 as well as $\dot{V}(k)\leq 0$, shows that V(k) has a finite value, i.e., $t\to \infty \Rightarrow \dot{V}(k)\to 0$,$\underset{t\to \infty }{\lim}\sum_{j=1}^{n}a_{ij}(\lambda_{i}(t_{i}^{k})-\lambda_{j}(t_{i}^{k}))=0$, written in matrix form as $\underset{t\to \infty }{\lim}L\lambda (k)=0$, when the communication topology graph is strongly connected, the Laplacian matrix L has an eigenvalue 0, corresponding to the eigenvector $1^{n}$, thus $\underset{k\to \infty }{\lim}\lambda_{j}(k)=\underset{k\to \infty }{\lim}\lambda_{i}(k)$, i.e., consistency is achieved and the proof is complete.

## 4. Numerical simulations and discussion of results

**Fig 2:**
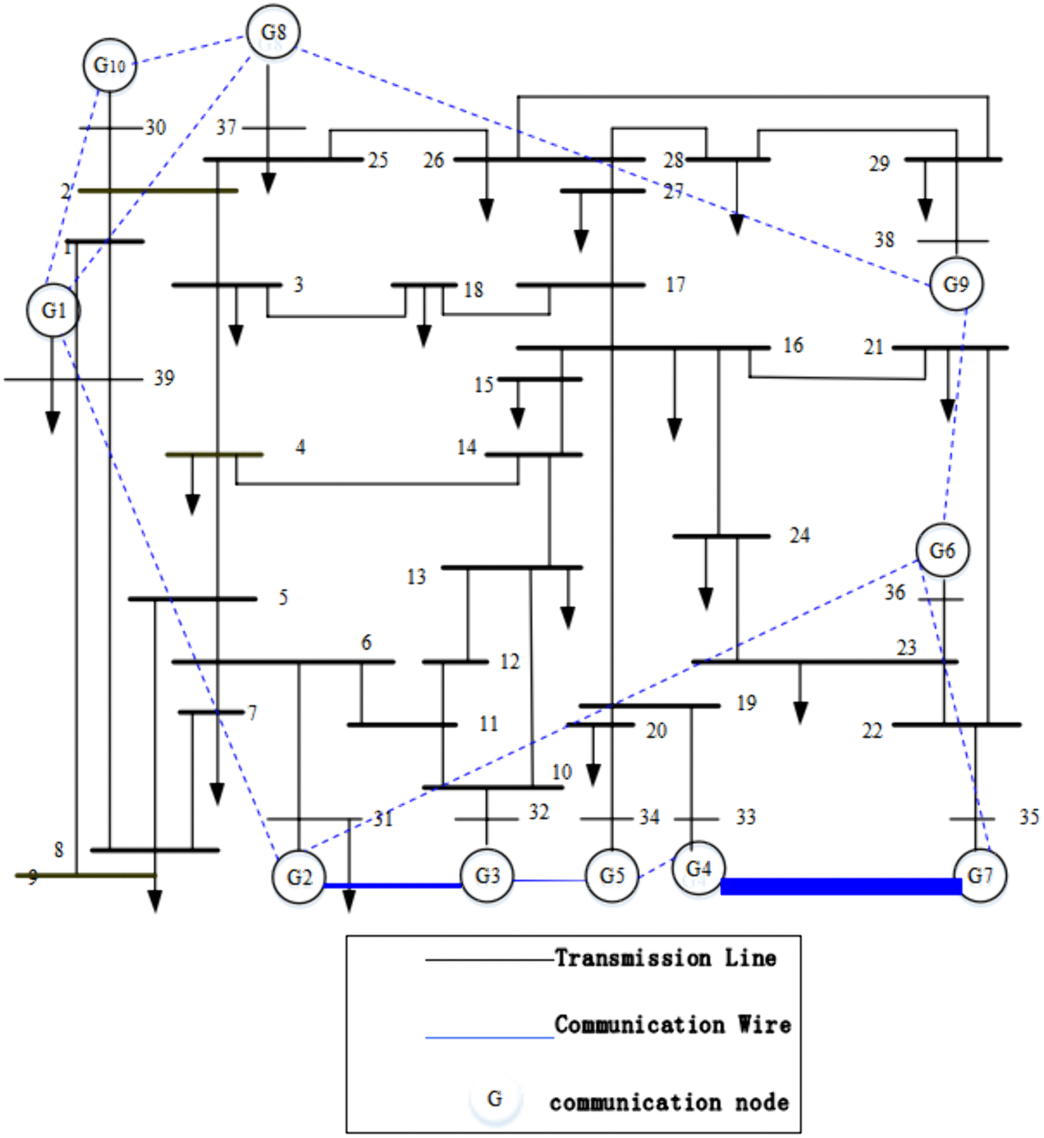
IEEE 39 Busbar System Diagram.

In this section, the effectiveness of the above proposed distributed economic dispatch algorithm based on event-triggered mechanism is verified by numerical simulation and analysis of the arithmetic example. The arithmetic example is simulated and analyzed using an IEEE 10-machine 19-load 39-node system, which consists of 10 generators and 19 loads, and the communication topology is given in blue dashed lines as shown in Fig 2, which shows that the communication topology is strongly connected and balanced. The parameters and initial conditions of all generators are shown in Table 1.

**Table 1:** 11-machine 19-load system parameters.

Electrical Machinery	$a_{i}$	$b_{i}$	$c_{i}$	$P_{Gi}(0)$ (Pressure)
1	300	7.92	0.001561	200
2	250	7.85	0.00194	250
3	560	7.8	0.00482	100
4	160	7.92	0.001561	200
5	561	7.8	0.00482	100
6	540	7.9	0.00204	150
7	230	7.5	0.00145	100
8	150	7.4	0.00256	120
9	210	7.6	0.00356	180
10	170	7.59	0.00247	200

**Fig 3:**
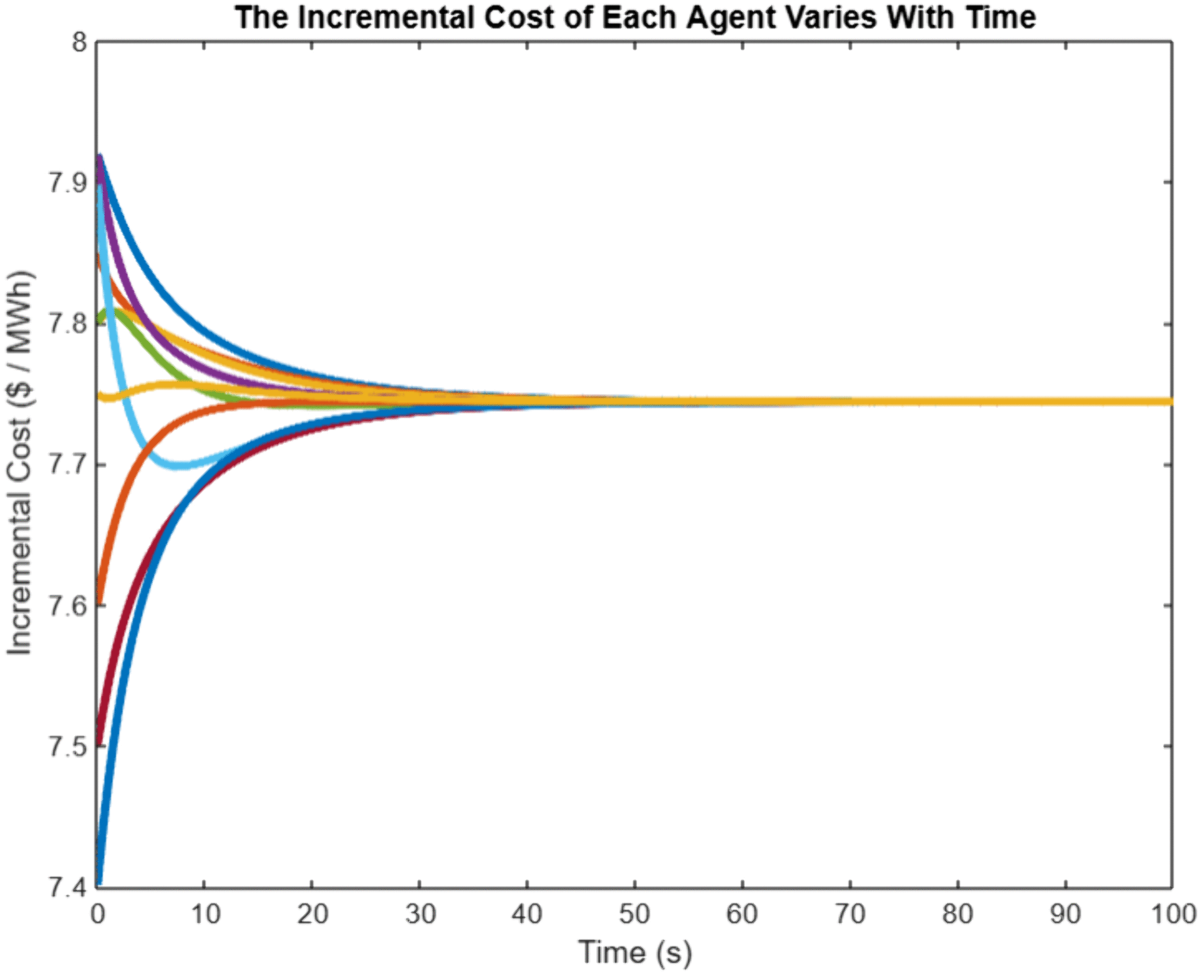
Incremental cost variation chart for each intelligent agent.

**Fig 4:**
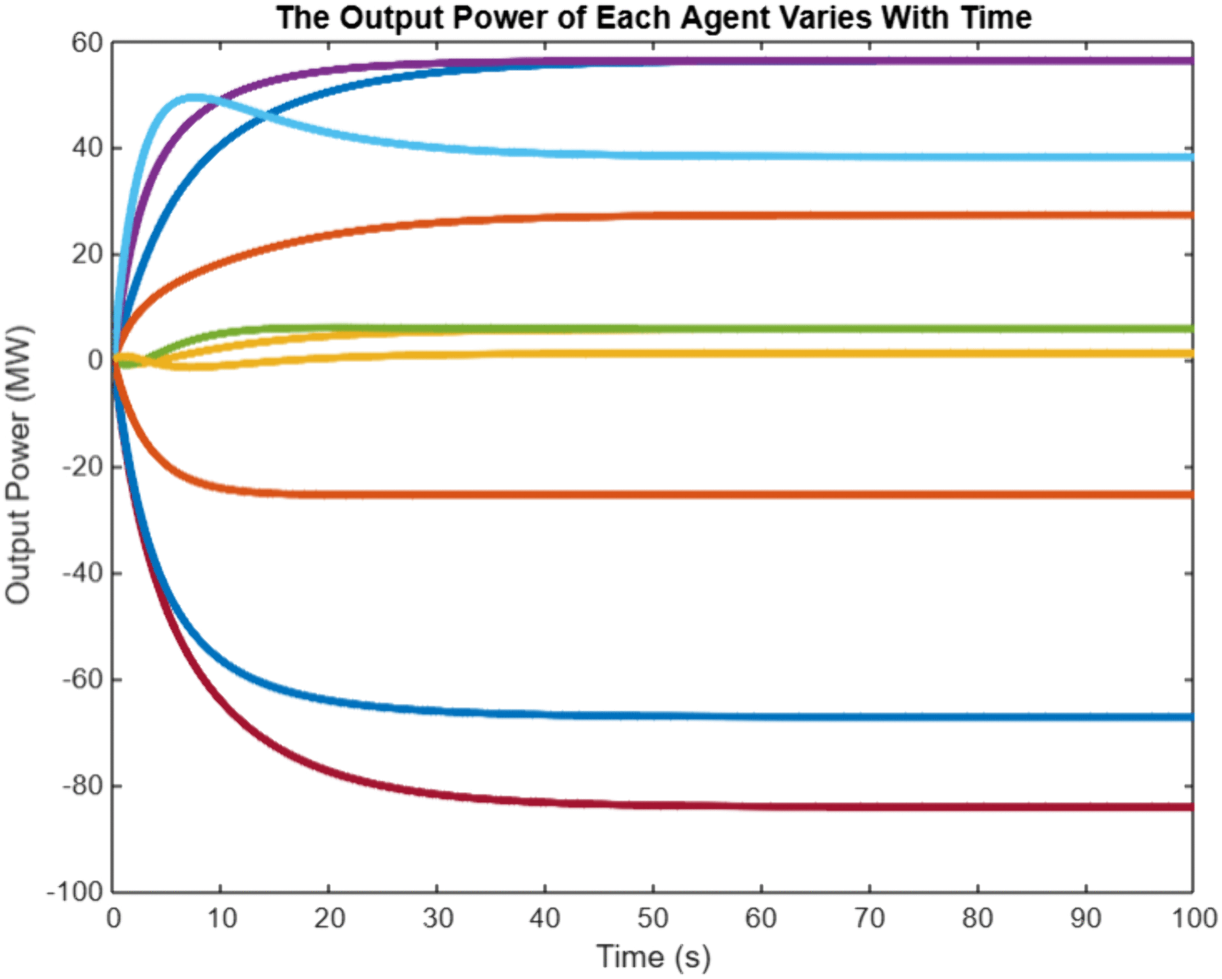
Power output variation diagram for each intelligent agent.

In order to verify the effectiveness of the proposed algorithm based on the event triggering mechanism, G10 is selected as the Leader generator, in which the sampling step is 0.001-s, and the convergence coefficients are set at the same timeε=0.005,β=0.95, the incremental cost of the generators $\lambda_{i}$, the output power $P_{Gi}$ and the total output power of the system Simulation results are shown in Fig 3, and the triggering intervals of the 10 generators are shown in Fig 3, in which the The horizontal coordinate of the ‘o’ sign represents the trigger time, and the vertical coordinate represents the time interval between the current trigger and the last trigger. From Fig 3, it can be seen that the incremental costs of all the generators in the system eventually converge to the same value, which achieves the objective to be optimized, and from Fig 3, it can be seen that the triggering moments of each generator are discrete, and they are triggered with unequal periods As shown in Fig 4, the algorithm based on event triggering mechanism proposed in this chapter can decide whether to execute the control tasks according to the pre-set triggering conditions, realizing the “on-demand execution”, avoiding the transmission of redundant information in the network, and reducing the pressure of network transmission. The system consistency variable,$\lambda^{*}=7.744$, is the same as that calculated by the centralized method, which also meets the power balance constraints and verifies the effectiveness of the proposed algorithm.

**Fig 5:**
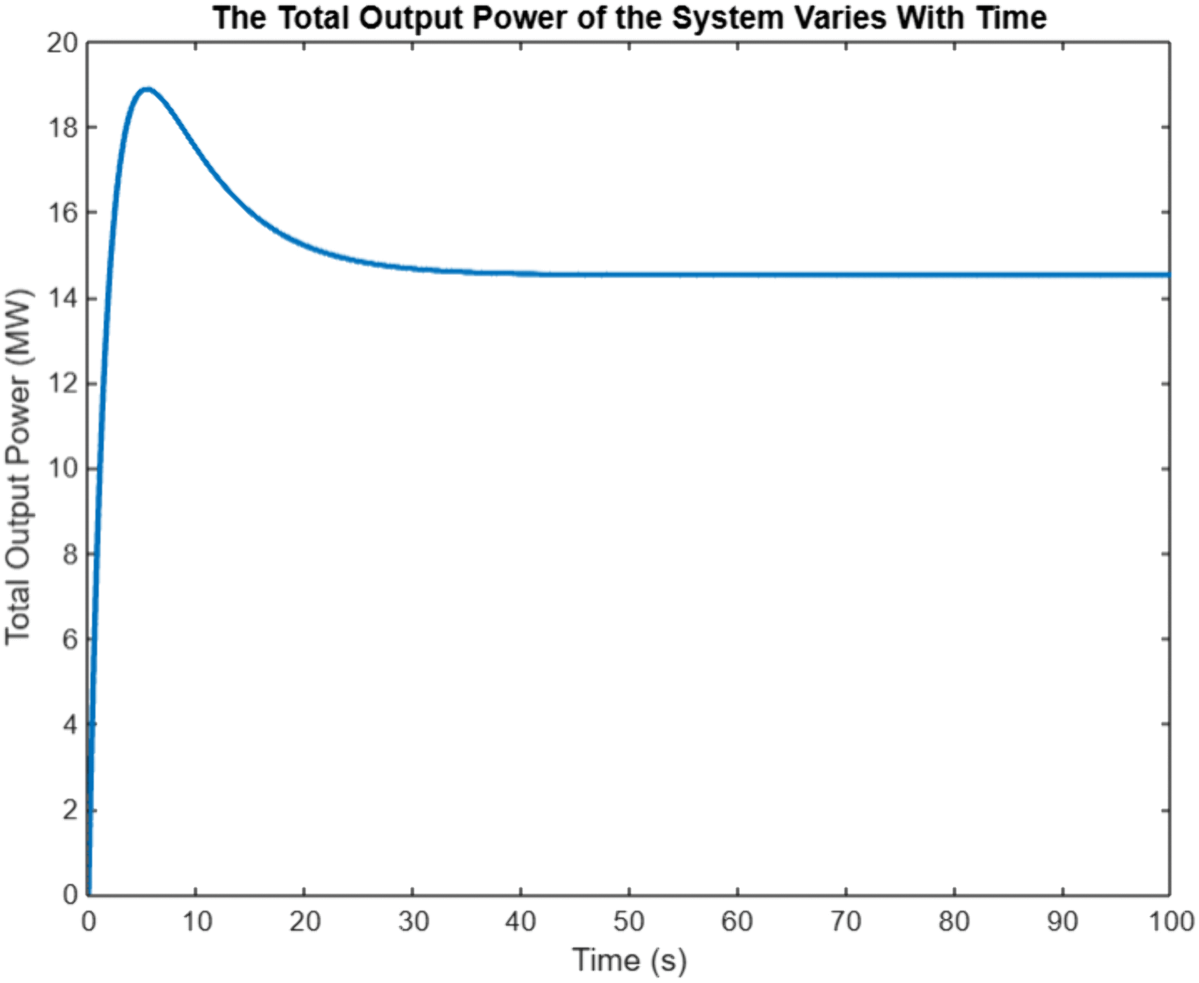
System total output variation chart.

In the power regulation process of HVAC multi-agent system, when there is a mismatch between the preset total load demand and the actual total reduction, that is Δp≠0, the initial supply and demand deviation will act as a dynamic compensation factor on the global power correction. Based on the multi-agent collaborative optimization framework, the power distribution balance of each load node is realized through iterative information interaction, which follows the convergence process shown in Fig 3 and Fig 4. Under this distributed cooperative control mechanism, each agent dynamically updates the power setting value according to the neighborhood information until the incremental cost consistency condition is satisfied. Numerical analysis shows that with the gradual convergence of power allocation, the incremental cost parameters show synchronous convergence characteristics, and the specific dynamic evolution trajectory is shown in Fig 5. It can be seen from the figure that the system can achieve power balance within 60 seconds. The synchronous convergence characteristics of incremental cost verify the effectiveness of the algorithm and provide a theoretical basis for the construction of HVAC cooperative control system.

**Fig 6:**
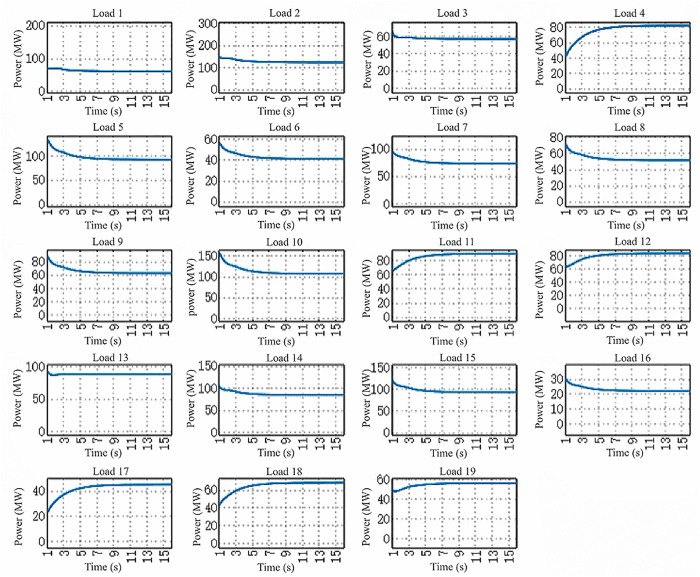
Evolution Curves of Air Conditioning Load Reduction for 19 Scenarios.

Fig 6 illustrates the evolution curve of load reduction across 19 nodes in the HVAC system. Analysis of simulation results reveals that each load exhibits distinct dynamic characteristics within the 1–16 second observation window. The overall system demonstrates significant hierarchical

response characteristics: approximately 75% of loads (e.g., Loads 1–8, 13–15) exhibit power fluctuations below ±10%, maintaining within ±15MW of the baseline value, indicating good steady-state properties for this portion of the load. In contrast, nodes such as Loads 9, 10, and 12 exceeded 40MW power steps between t = 5–8 seconds, with maximum instantaneous fluctuation rates reaching 32.8%, revealing transient responses to control strategies. Power reductions across all loads stabilized after 15 seconds, validating the algorithm’s effectiveness and providing a basis for dynamic grid stability analysis and distributed control optimization.

**Fig 7:**
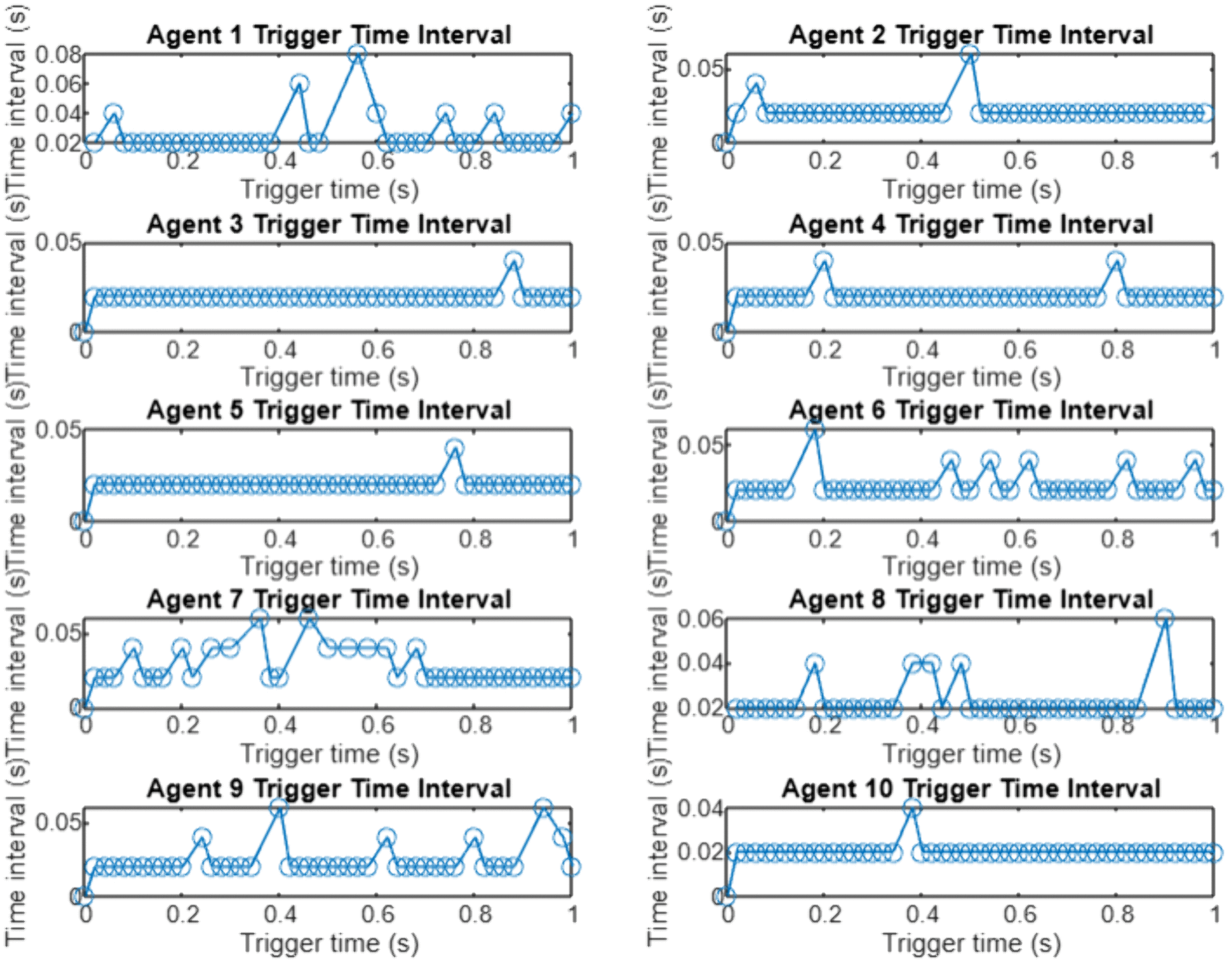
Trigger intervals for each generator.

In this study, a simulation verification platform containing 10 generating units was constructed, and the G10 unit was selected as the leading coordination node and the consistency protocol was configured. The experiment uses a discrete-time simulation architecture, sets the time step, convergence factor,. Under the dynamic event triggering mechanism, each distributed node updates the state information according to the preset threshold condition, and its communication timing distribution is shown in Fig 7.

All the consistency variables in the system are still consistent and the power balance constraints are satisfied. The trigger time interval of each generator satisfies the discrete non-equal period. It shows that the algorithm based on the event trigger mechanism proposed in this chapter can determine whether the control task is executed according to the preset trigger conditions, realize ‘ on-demand execution ‘, avoid the transmission of redundant information in the network, and reduce the pressure of network transmission.

## 5. Conclusion

From the perspective of distributed optimization, this paper proposes a new distributed control algorithm based on event-triggered mechanism. By designing a trigger control condition to control the task ‘ on-demand execution ‘, while maintaining performance, it can avoid redundant information transmission in the network, reduce the pressure of network transmission, and save computing resources. The scheduling model is described by a quadratic convex cost function. The communication topology adopts a strong connected graph, so that the information is fully transmitted between the power components. The control method used does not require global state information, only local and neighbor information. Finally, the incremental cost of the desired power generation unit is consistent, and all the generators work together to achieve cost optimization under the condition of satisfying the power balance constraint. The simulation results show that the consistency algorithm based on the event trigger mechanism has good robustness and convergence. At the same time, it greatly reduces the information transmission pressure between the power generation units, effectively solves the constraints of limited communication network bandwidth, and ensures the stable and safe operation of the power system.This paper focuses on the energy-saving optimisation of building heating, ventilation and air conditioning (HVAC) systems, conducting systematic research centred on theoretical innovations and engineering applications of multi-agent collaborative control and event-triggered mechanisms. The research encompasses mathematical modelling, algorithm design, theoretical proof and simulation validation, specifically structured across the following three levels:

(1) Theoretical Foundations and Collaborative Control Framework Construction.For first-order and second-order multi-agent systems, consistent control architectures have been proposed for both continuous-time and discrete-time frameworks. These theoretical achievements lay the mathematical foundation for subsequent development of event-triggering mechanisms.
(2) Innovation in Event Triggering Mechanisms and Performance Optimisation.To address the strong non-linear characteristics of HVAC systems, a dynamic threshold adaptive adjustment strategy is proposed: linking trigger conditions to the system’s real-time operational state to achieve a dynamic equilibrium between communication efficiency and control precision.
(3) Practical Implementation of Distributed Energy-Saving Optimisation for HVAC Systems.To address the issue of constrained communication bandwidth, a dynamic threshold event-triggering mechanism is proposed. By constructing discrete-time Lyapunov functions and employing matrix perturbation theory, it is demonstrated that under strongly connected topologies, the IC and IB values of all agents can converge to globally consistent values within finite time.

## 6. Future work directions

Due to the many shortcomings that still exist in my professional research.

(1) This paper validates the algorithm’s effectiveness solely through simulation experiments, yet fails to adequately account for the impact of complex factors such as sensor noise and device heterogeneity in real-world building environments.
(2) This paper employs a fixed-threshold design for the event-triggering mechanism,however, in practical scenarios, sudden load fluctuations or dynamic changes in the communication topology may lead to a deterioration in convergence performance.
(3) This paper focuses solely on energy consumption optimisation objectives, whilst neglecting multi-objective coordination requirements such as user comfort (e.g., PMV index) and equipment lifespan.
