# Correction: Research on energy-saving algorithm of HVAC multi-agent system consensus based on event-triggered mechanism

**DOI:** 10.1371/journal.pone.0344626

**Published:** 2026-03-09

**Authors:** Weilin Wu, Shaoxiong Shi, Meihuan Lin, Haixiao Gong, Jiakan Li

There are errors in the author affiliations. The correct affiliations are as follows:

Weilin Wu^1^, Shaoxiong Shi^1^, Meihuan Lin^1^, Haixiao Gong^2^, Jiakan Li^3^

**1** Center for Applied Mathematics of Guangxi, College of Physics and Electronic Information, Guangxi Minzu University, Guangxi, China, **2** Guangxi Key Laboratory of Machine Vision and Intelligent Control, Wuzhou University, Guangxi, China, **3** Guigang Information and Government Service Center, Guangxi, China.
